# Generation of Oxalic Acid in Syrup of Sesqui-Nitrate of Iron

**Published:** 1853-03

**Authors:** 


*Generation of Oxalic Acid in Syrup of Sesqui-Nitrate of Iron.*—Mr. J. LAIDLEY, of Richmond, Va., has shown, in an interesting paper in the last number of the American Journal of Pharmacy, that the *syrup* of *sesqui*-nitrate of iron is an unscientific and ineligible preparation; for, without an excess of acid, it is a mixture of proto and per-nitrate, and with that excess the acid generates oxalic acid. When a syrup of nitrate of iron is wanted, the *proto*-nitrate should be employed; in it there is no excess of acid to act on the sugar, which is employed to prevent peroxidation of the iron, and it is not only permanent but pleasant to the palate.

